# Anterior segment mesenchymal dysgenesis in a large Australian family is associated with the recurrent 17 bp duplication in *PITX3*

**Published:** 2008-11-05

**Authors:** Kim M. Summers, Stephen J. Withers, Glen A. Gole, Sara Piras, Peter J. Taylor

**Affiliations:** 1 The Roslin Institute, The University of Edinburgh, Roslin, Midlothian, UK; 2 School of Molecular and Microbial Sciences, The University of Queensland, Brisbane, Queensland, Australia; 3 Genesis Clinical Genetics, Southport, Queensland, Australia; 4 Eye Department and Discipline of Paediatrics and Child Health, The University of Queensland, Royal Children’s Hospital, Brisbane, Queensland, Australia; 5 Molecular and Cytogenetics Unit, Prince of Wales Hospital, Sydney, New South Wales, Australia

## Abstract

**Purpose:**

A recurrent 17 bp duplication (c.657ins17bp) of a segment of the paired-like homeodomain transcription factor 3 (*PITX3*) gene on human chromosome 10 has been reported in seven families with autosomal dominant posterior polar cataracts with or without anterior segment mesenchymal dysgenesis (ASMD). ASMD can include Peters anomaly with corneal clouding, iridolenticular corneal adhesions, displaced Schwalbe’s line, and cataract as described previously in a large Australian family. This study reports the examination of *PITX3* in this Australian family.

**Methods:**

Clinical examinations of the proband and her relatives were performed as part of routine follow up. A polymerase chain reaction (PCR) based test for the duplication in *PITX3* was developed, and DNA from 21 members of the proband’s family was tested.

**Results:**

All clinically affected members of the family had the same 17 bp duplication of *PITX3*. There was no difference in the size of the duplication between the severely affected individuals and the more mildly affected individuals. Prenatal diagnosis was performed for two offspring of one affected person. In the first pregnancy, the fetus was shown to carry the duplication while in the second pregnancy, the fetus was shown to be homozygous for the normal allele.

**Conclusions:**

The results show that in some individuals within one family, duplication of this segment of *PITX3* can result in severe symptoms leading to functional blindness while in other individuals in the same family or in other families, the same duplication leads to treatable cataract with minimal visual impairment.

## Introduction

The term, Peters anomaly, describes an abnormality of the embryonic development of the anterior segment of the eye [1]. Affected individuals may have corneal clouding and displaced Schwalbe’s line; adhesions between the cornea, iris, and lens; and early development of cataracts. This complex of ocular signs has been called anterior segment mesenchymal dysgenesis (ASMD) [2]. We have described a four-generation family with variable expression of ASMD, from one individual with clouding of both corneas resulting in blindness, to several family members with bilateral early onset cataracts only. In total, seven individuals had some signs of ASMD while eight had cataract as the only manifestation [3]. Cataracts were detected as early as one year of age and were extracted between the ages of 8 and 38 years. The condition in this family was considered to be inherited in an autosomal dominant fashion. We noted that the lack of male to male transmission was also consistent with X-linked dominant inheritance, although there were very few males at risk [3].

Peters anomaly has been associated with abnormalities of several genes that have a role in the development of the anterior segment of the eye (Table 1). We ruled out linkage with a segment of the paired box 6 (*PAX6*) gene in our family [3] and could not find linkage with chromosome 4 markers (unpublished). A 17 bp duplication of a segment of the paired-like homeodomain transcription factor 3 (*PITX3*) gene was initially discovered in a large family with ASMD [4]. More recently, the same duplication has been reported in six families with posterior polar cataract [5-7]. A single base deletion in the same gene has also been seen in a posterior polar cataract family [5]. Very few affected individuals in these families (5/106) had ASMD [5-8], indicating that cataract is the major manifestation of the mutations in this gene [5].

**Table 1:** Genes associated with Peters anomaly and anterior segment mesenchymal dysgenesis.

**Gene name**	**Protein function**	**Chromosome location**	**Major phenotype associated with mutations**	**Inheritance of ASMD**	**Reference for ASMD**
*FOXE3*	Transcription factor with fork-head DNA binding domain	1p32	ASMD (aphakia when homozygous or compound heterozygous)	Dominant	[12]
*CYP1B1*	Monomeric mixed function mono-oxygenase, dioxin responsive	2p22-p21	Congenital glaucoma	Recessive	[13]
*PITX2/RIEG1*	Transcription factor with bicoid paired DNA binding domain	4q25	Axenfeld-Rieger syndrome (ocular, dental and umbilical abnormalities)	Dominant	[14]
*FOXC1*	Transcription factor with forkhead DNA binding domain	6p25	Spectrum of ocular and extraocular phenotypes including congenital glaucoma, Rieger anomaly, Axenfeld anomaly, cardiac abnormalities	Dominant	[15]
*PITX3*	Transcription factor with bicoid paired DNA binding domain	10q25	Posterior polar cataract	Dominant	[4]
*PAX6*	Transcription factor with paired type DNA binding domain	11p13	Aniridia	Dominant	[16]
*B3GALTL*	Glycosyl transferase	13q12.3	Peters Plus syndrome (ocular, facial, developmental and stature abnormalities)	Recessive	[17]

Causes of phenotypic variability in the families with this duplication have not yet been elucidated. One possibility is that more severely affected individuals have an amplification of the insertion, since duplication of the segment could predispose them to unequal crossing-over events at meiosis or to replication slippage. Consistent with this hypothesis, there has been increasing severity of the condition over several generations in some branches of the family we have studied [3], suggesting the phenomenon of anticipation (see [9] for a review). We now provide a clinical update and report analysis of *PITX3* in this Australian ASMD family.

## Methods

**Figure 1:**
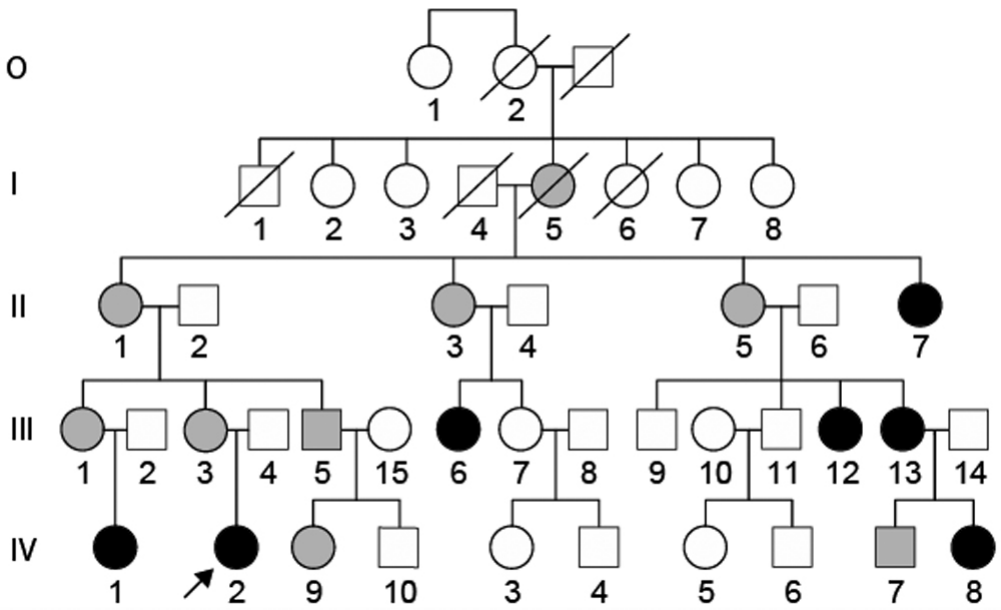
Pedigree of the anterior segment mesenchymal dysgenesis family. Clinical details are available in the previous paper [3]. Clinical details for IV-9 who has been born since that publication are presented in Methods. Grey symbols indicate individuals with cataract only, and black symbols indicate family members with cataract and additional anterior segment eye abnormalities. The proband, IV-2, is indicated with an arrow.

**Figure 2:**
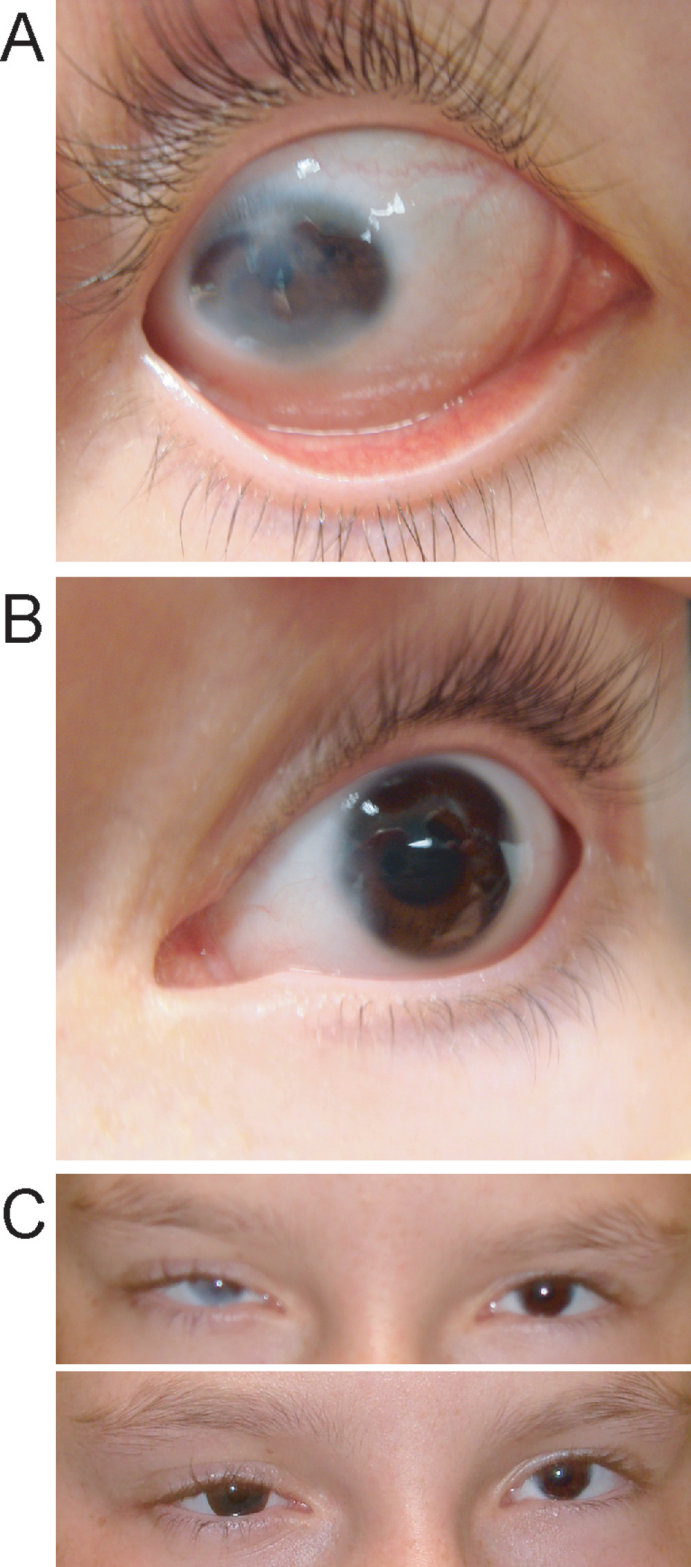
Proband, IV-2, at the age of 12 years and 6 months. **A** shows the severely affected right eye with peripheral sclerocornea. **B** shows the mildly affected left eye, with peripheral corneal  stromal opacity and displaced Schwalbe's line. **C** shows the proband's eyes without (upper) and with (lower) a colored contact lens in the right eye to mask the corneal opacity.

The study was approved by the Ethics Committee of the Royal Children’s Hospital in Brisbane, Australia. Informed consent was obtained from all adults and from the appropriate adult guardian for the children. Figure 1 shows the pedigree. Individual identification numbers are the same as in the previous paper [3] with the addition of the spouse and children of III-5 (III-15, IV-9, and IV-10). Results of detailed clinical investigations covering several years have been described [3]. At a recent investigation, the proband (IV-2) had hand movement vision in her right eye where severe corneal clouding has persisted from birth (Figure 2A). She had divergent strabismus of this eye, which was corrected with surgery at the age of four years for cosmetic reasons. The left eye had opacity of the peripheral cornea, displaced Schwalbe’s line, and iris adhesions (Figure 2B). Vision in the left eye was 6/18 at the age of 12 years and 6 months and 6/24 at the age of 14 years due to progressive posterior subcapsular cataract. At 12 years and 6 months, intraocular pressures were 33 mmHg in the right eye and 12 mmHg in the left (normal IOP ranges: 10–20 mmHg). She is currently wearing a colored contact lens in the right eye to mask the corneal opacity (Figure 2C). One affected individual, IV-9, was born since the last report. She was shown at the age of six months to have a small lamellar opacity in the left lens cortex, which did not progress over the next nine months. The right lens remained clear at 15 months of age. There were no other abnormalities of the anterior segment in this child.

**Figure 3:**
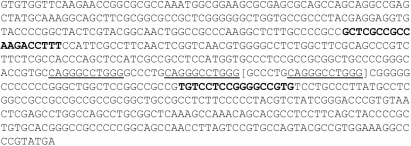
Sequence of exon 4 of *PITX3*. Primers are shown in bold. The 11 bp repeated sequence is underlined, and the 17 bp duplication within this block is enclosed in square brackets. The sequence terminates at the TGA stop codon.

DNA was extracted from venous blood samples as reported previously [3]. *PITX3* was screened by amplifying a 214 bp fragment of exon 4, the site of the 17 bp insertion in other families (Figure 3). The primers used were GCT CGC CGC CAA GAC CTT TC (forward) and CGA GGC ATA AGG GCA GGA CA (reverse). Reactions were performed in a final volume of 10 μl that contained 1.75 pmol of each primer, 1.5 mM MgSO_4_, 0.2 mM dNTPs, 3X PCRx Enhancer Solution (PCR, polymerase chain reaction; Invitrogen, Mt. Waverley, Victoria, Australia), 0.4 units Platinum Taq Polymerase (Invitrogen), 1X PCR amplification buffer (Invitrogen), and 100 ng genomic DNA. The amplification procedure consisted of an initial denaturation step of 4 min at 96 °C followed by 27 cycles of 30 s at 95 °C, 30 s at 58 °C, and 1.5 min at 72 °C. From cycle 11, the extension time at 72 °C was increased by 5 s each cycle. The amplification was completed with 7 min at 72 °C and 10 s at 26 °C. The ramp speed was 2 s/°C. Amplification products were run on 6% acrylamide gels (19:1; acrylamide:bisacrylamide) in 2X TAE buffer for 30 min and stained with silver staining to visualize the bands. The molecular weight marker was HpaII-digested plasmid, pUC19 (catalog number DMWP-1; Geneworks, Adelaide, South Australia, Australia).

## Results

**Figure 4:**
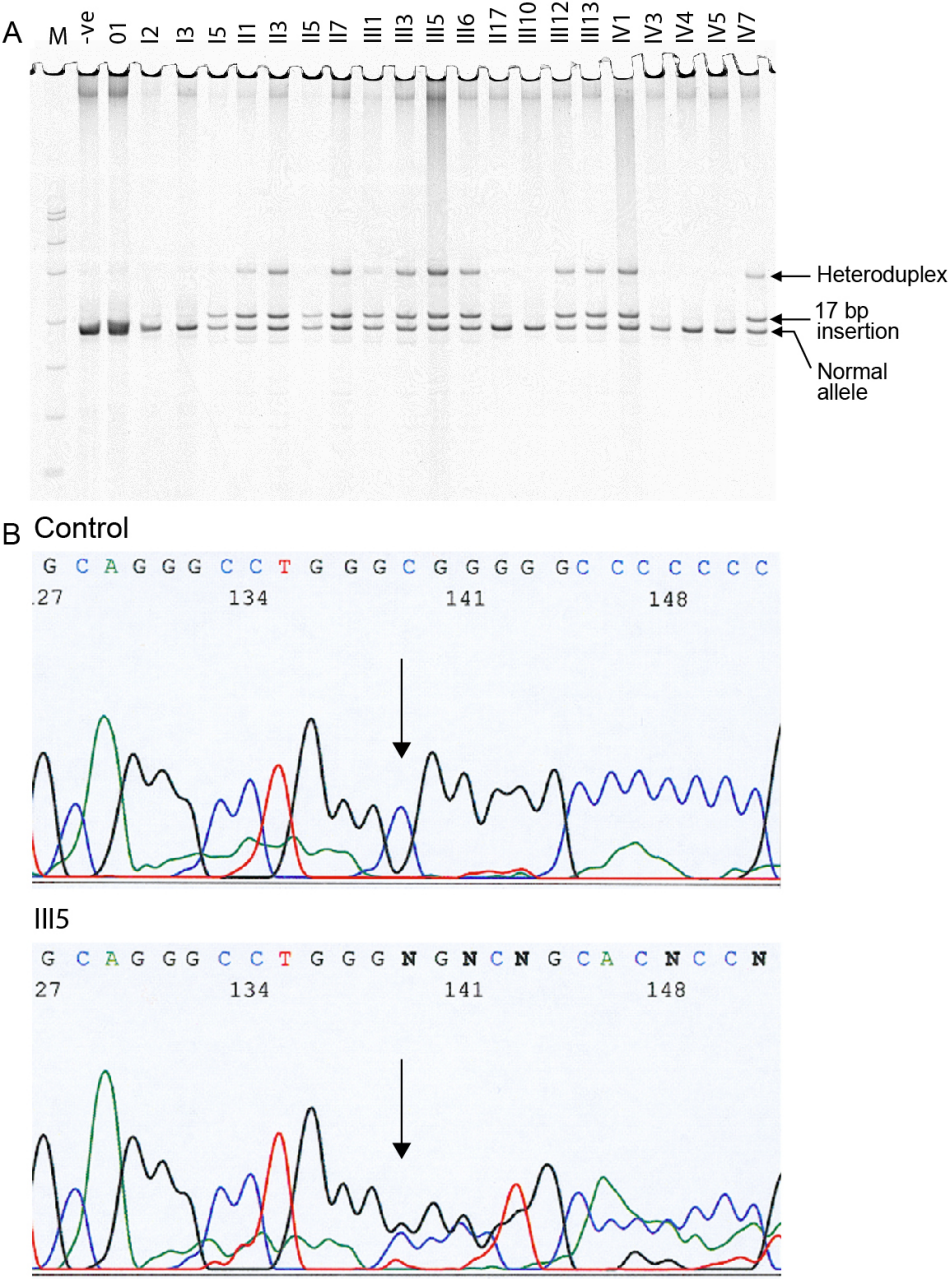
Analysis of exon 4 of *PITX3*.  **A**: The gel of amplification products is shown. The 231 bp band containing the insertion is clearly separated from the 214 bp band. A heteroduplex band can also be seen in heterozygous individuals. –ve stands for the unaffected, control individual. M stands for the molecular weight marker, a HpaII digest of the plasmid pUC19. The bands either side of the amplification products are 190 and 242 bp. The heteroduplex runs with the 331 bp band. **B**: The sequence of exon 4, containing the insertion, for individual III-5 is shown.

Initial attempts using the same primers as a previous study [4] failed to amplify exon 4 of *PITX3* in the DNA of members of this family. This could be due to the high GC content (67% GC) of the amplicon (Figure 3). The PCR protocol finally adopted (see Methods) allowed visualization of the DNA fragment following electrophoresis and silver staining. All tested individuals with cataract or manifestations of ASMD had amplicons of two sizes, 214 bp and 231 bp (Figure 4A), while all those with normal eye development had only one band, the smaller 214 bp fragment. There was also a larger heteroduplex band in the heterozygotes, which was missing from those without the duplication. DNA from one individual (III-5) was sequenced and showed the presence of the insertion on one copy of chromosome 10 (Figure 4B) similar to the result seen previously [4,5,7].

**Figure 5:**
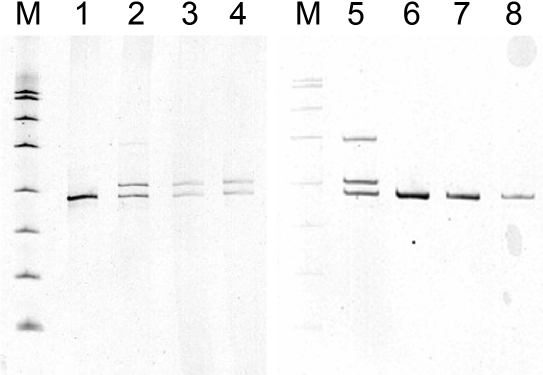
Prenatal diagnosis of the inherited duplication. Gels of amplification products are shown. Lanes 1 and 6 are a negative control. Lanes 2 and 5 are the positive control, III-5. Lanes 3 and 4 show two dilutions of the CVS material for IV-9. Lanes 7 and 8 show two dilutions of the CVS material for IV-10. M is the molecular weight marker, a HpaII digest of pUC19. IV-9 has the insertion band and IV-10 has only the normal band.

Prenatal diagnosis was performed for IV-9 and IV-10, the children of III-5, following chorionic villus biopsy. The result for IV-9 showed heterozygosity for the same insertion allele (Figure 5). The parents elected to continue the pregnancy, and a healthy child with no corneal abnormalities was born. A small cataract was observed at six months of age. The result for IV-10 showed only the normal band (Figure 5), and the baby was healthy with no corneal or lens abnormalities.

## Discussion

A study of Australian cataract patients [7] revealed only one family with the 17 bp insertion mutation in 101 families. We have now identified a second family with more extensive disease [3] in which the recurrent 17 bp duplication of *PITX3* (c.657ins17bp) segregates with ASMD. Family members in our study have no knowledge of any relatives living in the same area of the country as the other family, suggesting that the mutations are independent. This is consistent with the different manifestations of anterior segment abnormalities in the two families and the probable de novo origin of the mutation in individual I-5 [3].

The protein encoded by this gene controls the early stages of normal development of the eye of mammals and other vertebrates [10,11]. It functions as a transcription factor, regulating the expression of genes that act downstream of *PAX6* in eye development. Families with the *PITX3* insertion have no abnormalities in other organ systems, and it is likely that the PITX3 protein is exclusively involved in eye development. The variable phenotypic impact of the insertion (from cataract in most families to severe ASMD in some individuals) may be due to modifier loci, which could include any of the genes previously shown to harbor mutations in ASMD (see Table 1), or to environmental effects influencing eye development during gestation. All of the affected individuals in our family gave an identical fragment size for the insertion, ruling out the possibility that the more severe phenotype is associated with further amplification of the repeated sequence within the insertion.

In this family, the initial insertion may have occurred in the oldest case examined (individual I-5). There is no evidence that either of her parents was affected, and her two sisters and aunt were shown in this study not to have the insertion. Individual I-5 had cataracts but no other anterior segment abnormalities when examined at the age of 86 years. One of her daughters, II-7, is the most severely affected family member while three of her granddaughters and three of her great granddaughters have some signs of ASMD [3]. In most previously reported families, the duplication results in posterior polar cataract without other anterior segment abnormalities. While all affected individuals in our family manifest cataract, half have additional signs of more extensive disruption of eye development.

There is no male-to-male transmission of eye disease in this family. Consistent with this, individual III-5 has recently had an affected daughter (IV-9) and an unaffected son (IV-10). The identification of the insertion in *PITX3* rules out X-linked inheritance. As noted in the previous paper [3], there are very few at risk males and the majority of affected individuals are female (14 out of 16). The high number of female offspring in this family may also reflect a sex limited effect of *PITX3*, which results in disproportionate loss of affected male pregnancies. However, the two affected males, III-5 and IV-7, have mild manifestations (cataracts only), which suggests there is no increased severity in males carrying the insertion.

Genetic counseling for this family is difficult because an individual with relatively minor effects (for example I-5) can have a severely affected child (II-7). However, in most family members, the condition is not debilitating and they achieve good visual acuity after cataract removal and intraocular lens insertion. For those individuals with corneal clouding, simple measures can be implemented such as the colored contact lens matching the unaffected eye now worn by IV-2, which provides cosmetic improvement and allows her to avoid stigmatization because of her different eyes.

Chorionic villus samples from the pregnancies of III-5 and III-15 were tested for the presence of the insertion. The first fetus (IV-9) was shown to have the duplication. After consideration of possible outcomes, the parents who felt they had bonded strongly with the fetus elected to continue the pregnancy and to deal with the visual problems as they arise. The baby had no signs of ASMD at birth but has a small non-progressive opacity in one lens. Like her father, she will probably develop more extensive cataracts as she ages but is likely to retain good vision with the aid of cataract surgery. Testing of the second pregnancy showed an unaffected fetus. The availability of this simple PCR-based test has allowed analysis of these pregnancies, but until the causes of phenotypic variability can be elucidated, the extent of the abnormality in any individual carrying the insertion cannot be predicted.
